# Comparison of the quadrant method measuring four points and bernard method in femoral tunnel position evaluation on 3-dimensional reconstructed computed tomography after anatomical single-bundle anterior cruciate ligament reconstruction

**DOI:** 10.1186/s12891-024-07678-6

**Published:** 2024-07-18

**Authors:** Jingxin Li, Jie Yang, Zhaoguang Xu, Weican Wang

**Affiliations:** 1 Xiamen Hospital of Traditional Chinese Medicine, 1739 Xianyue Road, Huli District, Xiamen City, 361015 China; 2 Department of Ultrasound Medicine, Zhongshan Hospital of Xiamen University, School of Medicine, Xiamen University, 201-209 Hu Bin South Road, Siming District, Xiamen City, 361004 China

**Keywords:** Anterior cruciate ligament reconstruction, Femoral tunnel position, 3-Dimensional computed tomography, Quadrant method

## Abstract

**Purpose:**

This prospective study aimed to compare the postoperative evaluation of the quadrant method measuring four points and Bernard method in femoral tunnel position evaluation on 3-Dimensional (3D) reconstructed computed tomography (CT) following the arthroscopic single-bundle anterior cruciate ligament (ACL) reconstruction.

**Methods:**

Thirty-eight patients with ACL tears that were reconstructed using single-bundle ACL reconstruction between May 2021 and March 2023 were included in this study. Postoperative 3D CT images were obtained after the operation. The femoral tunnel position was measured by use of the quadrant method measuring four points and Bernard method.

**Results:**

Average mean position of the femoral tunnel insertion center on the 3D CT image was at 26.16 ± 6.27% in the x-coordinate and at 24.36 ± 5.52% in the y-coordinate according to the Bernard method. Meanwhile, the position of the femoral insertion of the ACL measured by the quadrant method measuring four points was 24.2% ± 6.86% in the x-coordinate and 21.16% ± 5.14% in the y-coordinate.

**Conclusions:**

Both the quadrant method measuring four points and Bernard method were effective in femoral tunnel position evaluation on 3D reconstructed CT. Application of the quadrant method measuring four points on 3D CT showed the advantage that measurement can be taken regardless of the shape of the bone tunnel.

## Introduction

Anterior cruciate ligament (ACL) rupture are frequent injuries, and ACL reconstruction is the undisputed surgical technique of choice [1]. The main purpose of ACL reconstruction is to restore the original shape of the ACL, restore the anatomical attachment point, so that the reconstructed graft remains the same length during knee extension and flexion, thereby replacing the injured ACL [2]. Tunnel position influences tibial anterior translation and rotation and postoperative clinical outcomes. Tunnel malposition causes recurrence of knee instability and increased risk of osteoarthritis after ACL reconstruction [3]. It is of significance to select an appropriate method to observe the starting and ending points of the tibia and femur of the reconstructed ligaments and the angle of the bone tunnel, so as to provide an objective basis for evaluating the path of establishing the surgical bone tunnel.

The Bernard quadrant method is the most commonly used method for postoperative evaluation of ACL reconstruction [4]. This method has been verified in CT scan analysis [5, 6]. Tunnel location is often difficult to evaluate on a plain radiograph and, therefore, computed tomography (CT) is more commonly used to provide better visualization of bony structure [7]. The quadrant method measuring four points shows more reliable and detailed results in the evaluation of anatomical double-bundle ACL reconstruction according to the study of Yuta Mochizuki et al. [7]. Therefore, we hypothesized that the combination of these two methods may be more accurate for monitoring positions of femoral sites. In this study, the quadrant method measuring four points method was used to evaluate femoral tunnel position after anatomical dingle-bundle ACL reconstruction by measuring the highest, deepest, lowest and shallowest points of the femoral tunnel location with 3-dimensional (3D) CT, and the depth and height values of each point were measured.

We aimed to compare the Bernard quadrant method and the quadrant method measuring four points for postoperative evaluation of single-bundle ACL reconstruction.

**Table 1:** Measured value of the average of femoral tunnel positions by two methods

	Four-point quadrant method	Quadrant method	*P* value
Depth (in the x-coordinate)	24.20 ± 6.86%	26.16 ± 6.27%	0.580
Height (in the y-coordinate)	21.16 ± 5.14%	24.36 ± 5.52%	0.852

## Methods

Thirty-eight patients who underwent anatomic single-bundle ACL reconstruction between May 2021 and March 2013 were enrolled in this study. The patients were 28 males and 10 females, with an average age of 33 years (15–58 years) at the time of surgery. The information of hospital records including surgical notes and postoperative radio graphs were collected. The inclusion criteria were: (i) patients had subjective instability and functional impairment confirmed by a positive Lachman test and/or pivot-shift test result; (ii) ACL lesions confirmed by MRI and intraoperative arthroscopy; (iii) patients who were scheduled and underwent single-bundle ACL reconstruction with a hamstring autograft; (iv) an experienced surgeon participated as an operator. The exclusion criteria were: (i) patients had history of surgery on affected knee; (ii) the affected knee suffered from moderate to severe arthritis; (iii) patients who had prior intra-articular or extra-articular malalignment; patients had moderate or severe osteochondral degeneration on radio-graphic examination.

**Fig. 1:**
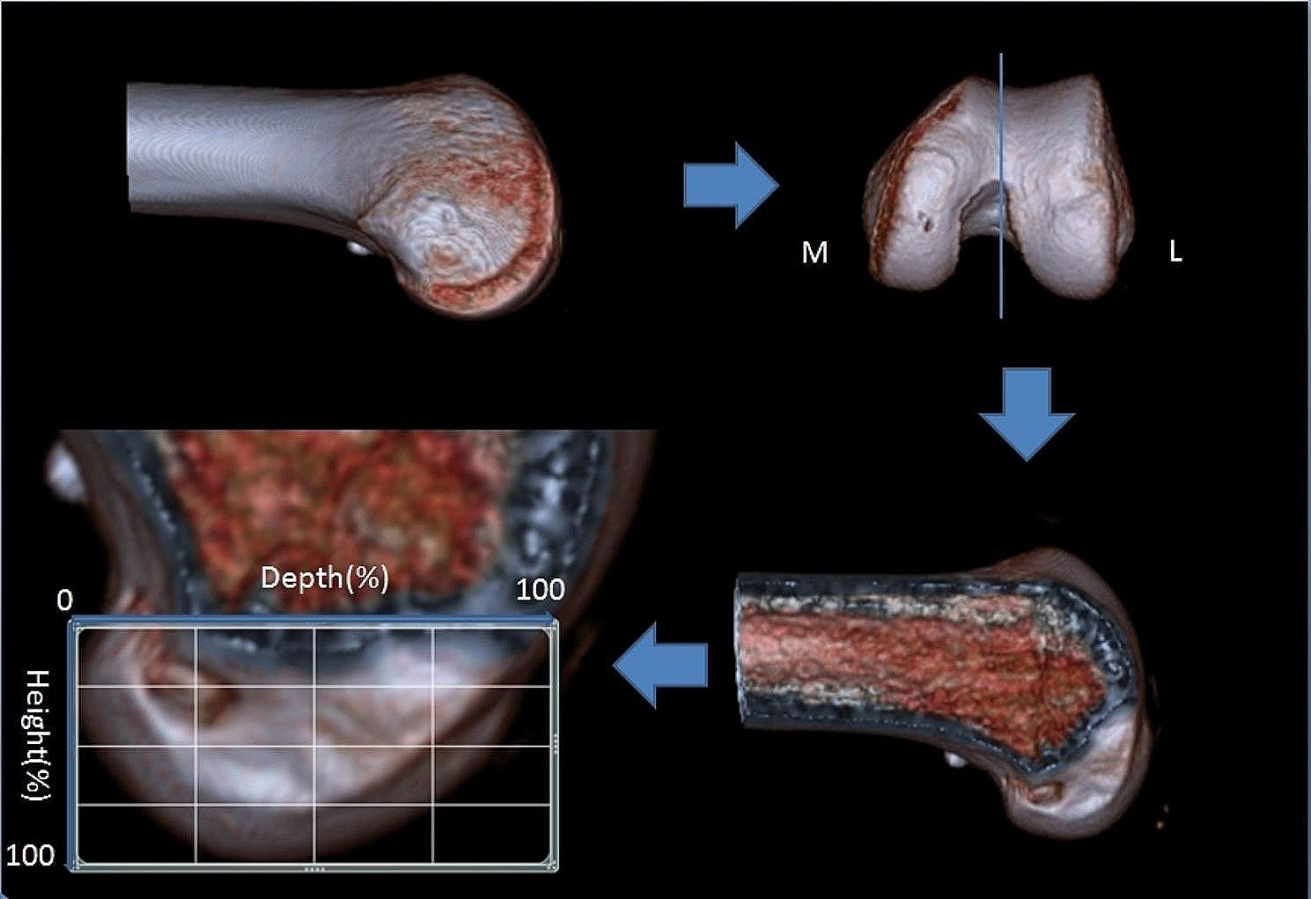
The M–L view of thelateral femoral condyle was obtained from the 3D CT of the distal femur in the strictly lateral position, where both condyles were superimposed. 3D CT of the medial–lateral (M–L) view was applied to 4 by 4 grid based on the quadrant method. M–L, medial–lateral

3D CT photos of all knees were taken after the surgery with the GE Light speed 4-slice scanner (GE Medical Systems). The parameters of CT scan were set as follows: helical mode, 0.625-mm-thick slices, 0.625 incrementation, bone and standard algorithm. Data were imported to PACS software (PACS 1 2.2, Philips) to create a 3D model of each distal femur. According to the Bernard method, the top of the intercondylar fossa was clearly shown at the optimal position of the bone tunnel on the 3D CT reconstruction. A snapshot of the medial-lateral (M-L) view of the lateral femoral condyle was taken in this lateral position. Finally, the image of 3D CT was downloaded and Microsoft PowerPoint for Macsoftware was used to create the rectangular outline. The boundaries of the rectangular outline are defined as follows: upper boundary: the highest point of the apex of the femoral intercondylar notch; inferior boundary: the level of the line parallel to the upper boundary at the posterior margin of the lateral femoral condyle; the superficial and deep boundaries are the junction of the distal and proximal condyles of the lateral femur (Fig. 1). The measurement was repeated after a 4-week interval by both observers.

**Fig. 2:**
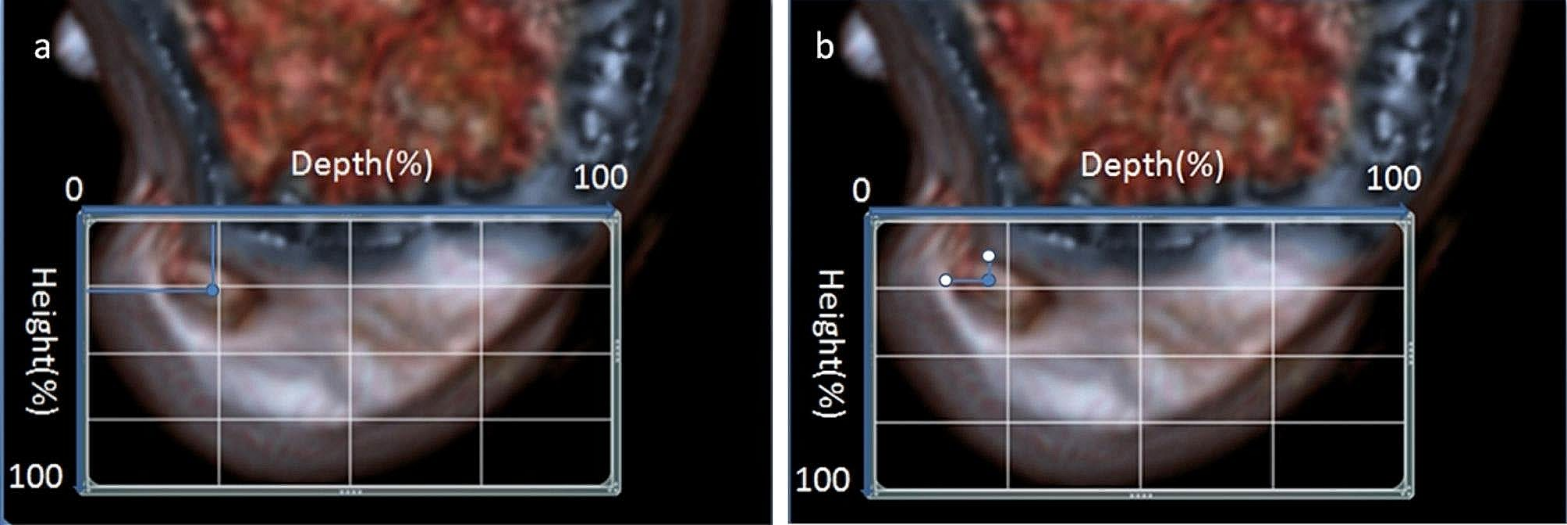
Measurements of femoral tunnel positioning **a** The quadrant method (described by Bernard et al.), **b** Four-point quadrant method

Lateral view images of the lateral condyle of the femur were applied to a 4 × 4 grid at this lateral location, and the femoral tunnel location was measured using two different methods. Then the Bernard quadrant method was used to measure 3D reconstructed CT after anatomical single-bundle ACL reconstruction. The percentage of the grid described from the posterior condyle and Blumensaat lines was then measured against the center of the anterior cruciate ligament bundle (Fig. 2A). At the same time, we measured the highest and deepest points of the femoral tunnel by four-point quadrants. The ratio of the value of the intersection of two points measured by the quadrantal method to the depth and height is obtained (Fig. 2B). To compare the value of depth and height of each point with the quadrant method and the four-point quadrant method, the independent-samples t-test was used to analyse continuous variables. All of the contrasts were considered significant when *p* < 0.05. Statistical analysis was performed by using SPSS software (version 12.0).

## Results

As indicated in Table 1, average mean position of the femoral tunnel insertion center on the 3D CT image was at 26.16 ± 6.27% in the x-coordinate and at 24.36 ± 5.52% in the y-coordinate according to the Bernard method. Meanwhile, the position of the femoral insertion of the ACL as measured by the quadrant method measuring four points was 24.2% ± 6.86% in the x-coordinate and 21.16% ± 5.14% in the y-coordinate. There were no significant differences between the two methods.

## Discussion

ACL injury is the most common type of knee ligament injury, and ACL reconstruction is needed after injury. The accurate positioning of the tibial tunnel can effectively avoid the complications of anterior knee pain, extension limitation, instability and graft impingement [8, 9]. The incorrect position of the internal opening of the tibial tunnel is one of the most common cases in poor arthroscopic ACL reconstruction [10, 11]. The internal opening of the tibial tunnel is too far forward, which can cause the graft to collide with the top of the intercondylar fossa, and the graft to relax or fracture. If the tibial tunnel is positioned forward, the graft will not only be subjected to excessive tension during knee flexion, but also may be impinged and damaged by the femoral condyle when the knee is extended. If the internal tibial opening is positioned too far back, the knee joint may appear relaxed and unstable even if the reconstructed ligament remains intact. If the tibial tunnel is positioned backward, the graft will appear too vertical and reduce the ability to restrict the movement of the tibia forward [12].

In patients with different therapeutic effects, the location of femoral tunnel was significantly different, indicating that the location of femoral shaft tunnel had a greater impact on postoperative joint function, and inappropriate femoral tunnel positioning had different effects on the isolength of ligaments [13]. If the internal opening of the femoral tunnel deviates from the isometric point, the graft will be subjected to great stress during knee extension and flexion, resulting in stretching and relaxation. If the femoral tunnel is too far down, too far forward, or both, the graft will be too vertical, resulting in reduced distance. When bending the knee, the tension on the graft will be significantly increased, which may cause the graft fiber to break and affect healing [14, 15].

Therefore, it is of significance to accurately pinpoint the femoral tunnel. However, there is no unified evaluation standard currently [16]. In this study, 3D reconstruction with volumetric CT scanning combined with clinical scoring was used to study the reconstructed anterior cruciate ligament to evaluate the surgical effect and prognosis. The femoral footprint of the native ACL was measured in various methods. The Bernard quadrant method was initially described as a radiological measuring method [17], which is done by obtaining a lateral radio graph of the knee with the femoral condyles super imposed. These representational methods all agreed that the central points of the ACL footprints should be the basis for the reference points used to obtain the arthroscopic view. Above all of the methods for evaluation ACL graft reconstructions of insertion techniques, each time an ACL reconstruction is performed, a center point has to be chosen for the tunnel and it makes sense that the center of the footprint would be a logical location for the center of the tunnel to be reamed. Regardless, the current trend is to reconstruct the ACL as close to the patient’s anatomy as possible, and it is assumed that the center of the ACL footprint is the best fit position. Since the ACL footprint is not a perfect circle [18–20], and the center of the footprint is not necessarily the ideal location for the reconstruction. In the four-point quadrant method, there is an advantage that measurement can be taken regardless of the shape of the femoral tunnel. However, we also found the four-point quadrant method is an equivalent method that has no significant differences and is as reliable as the Bernard quadrant method. The significance of this study is to explore whether the femoral tunnel localization evaluation method under CT detection is as reliable as the classical Bernard quadrant method, and to provide a demonstration for future comparison with other methods. Moreover, the evaluation methods of double-bundle and single-bundle reconstructed femoral tunnel can also be used the four-point quadrantal method can be used not only for the evaluation of the double-bundle ACL reconstruction, but also for the evaluation of the single-bundle ACL reconstruction.

## Conclusions

Both the quadrant method measuring four points and Bernard method were effective in femoral tunnel position evaluation on 3D reconstructed CT. Application of the quadrant method measuring four points on 3D CT showed the advantage that measurement can be taken regardless of the shape of the bone tunnel.

## Data Availability

The datasets used and/or analyzed during the current study are available from the corresponding author on reasonable request.

## Abbreviations

**Abbreviations**
3D: 3 Dimensional
CT: computed tomography
ACL: anterior cruciate ligament
M-L: medial-lateral
